# Long-Read Sequencing Unlocks New Insights into the *Amphidinium carterae* Microbiome

**DOI:** 10.3390/md22080342

**Published:** 2024-07-27

**Authors:** Miranda Judd, Jens Wira, Allen R. Place, Tsvetan Bachvaroff

**Affiliations:** Institute of Marine and Environmental Technology, University of Maryland Center for Environmental Science, Baltimore, MD 21202, USA; mjudd@umces.edu (M.J.); jwira@umces.edu (J.W.); place@umces.edu (A.R.P.)

**Keywords:** dinoflagellate, microbiome, long-read sequencing, secondary metabolites

## Abstract

Dinoflagellates are one of the largest groups of marine microalgae and exhibit diverse trophic strategies. Some dinoflagellates can produce secondary metabolites that are known to be toxic, which can lead to ecologically harmful blooms. *Amphidinium carterae* is one species of dinoflagellate that produces toxic compounds and is used as a model for dinoflagellate studies. The impact of the microbiome on *A. carterae* growth and metabolite synthesis is not yet fully understood, nor is the impact of bacterial data on sequencing and assembly. An antibiotic cocktail was previously shown to eliminate 16S amplification from the dinoflagellate culture. Even with drastically reduced bacterial numbers during antibiotic treatment, bacterial sequences were still present. In this experiment, we used novel Nanopore long-read sequencing techniques on *A. carterae* cultures to assemble 15 full bacterial genomes ranging from 2.9 to 6.0 Mb and found that the use of antibiotics decreased the percentage of reads mapping back to bacteria. We also identified shifts in the microbiome composition and identified a potentially deleterious bacterial species arising in the absence of the antibiotic treatment. Multiple antibiotic resistance genes were identified, as well as evidence that the bacterial population does not contribute to toxic secondary metabolite synthesis.

## 1. Introduction

Dinoflagellates are unicellular organisms that occupy multiple trophic levels in marine ecosystems, ranging from photoautotrophy, predation, mixotrophy, to even parasitism [1,2,3]. As significant components of phytoplankton communities, they produce a large portion of the world’s oxygen and serve as primary food sources for many marine organisms, such as zooplankton, small fish, and filter feeders [4,5,6]. Their ability to photosynthesize and sometimes feed enables them to thrive in various environmental conditions, making them vital for maintaining ecological balance and supporting biodiversity in aquatic habitats as both primary producers and consumers in the marine food web.

Some species of dinoflagellates are also known to cause harmful algal blooms (HABs), which can have devastating ecological and economic impacts [7,8,9]. These blooms occur when dinoflagellate populations rapidly grow under favorable conditions, producing toxins that can kill marine life and pose serious health risks to humans via seafood consumption or direct exposure. The toxins can also lead to significant economic losses in fisheries, tourism, and public health. On a broader level, the single-species dominance of a bloom influences the microbial community both directly and indirectly.

In the study of dinoflagellates, some species have shown to be culturable and are able to be kept in laboratory conditions in artificial seawater media. Many of these dinoflagellate cultures still contain abundant bacterial populations, likely originating from the original sample isolated from the environment [10,11,12]. The role of bacterial populations on dinoflagellate toxin production is one that remains hotly debated, with some prior studies showing evidence of dinoflagellate dependence on bacteria for toxin production and others showing the opposite [13,14,15,16,17,18]. Some of these studies involve the filtering of bacteria from dinoflagellate cultures, followed by null amplification of 16S regions as verification to prove that a culture is axenic, and subsequent high-performance liquid chromatography (HPLC) analysis to show whether a toxin is still produced in the axenic culture [19,20,21]. The results of these studies have been contradictory.

Currently, culturing dinoflagellates with antibiotics is a common practice to select organisms of interest and maintain monoclonal conditions. Eukaryotic cultures can be maintained with antibiotics to decrease the effects of bacterial populations on downstream analysis such as sequencing efforts, toxin analysis, and translation rate studies [22]. Although antibiotic use in dinoflagellate culturing can be useful, there is concern about bacterial antibiotic resistance allowing for regrowth of bacterial populations, as well as the impact antibiotics can have on the growth of the dinoflagellate culture.

We propose a new approach to investigate bacterial contributions to dinoflagellate cultures, by way of full bacterial genome assembly from long-read sequencing to identify pathways present in those bacterial populations, determine microbial taxonomic composition, and estimate relative abundance [23,24]. Here, we use this approach in a toxin-producing *Amphidinium carterae* CCMP1314 culture with a long history of laboratory growth in the presence of carbenicillin, kanamycin A, and spectinomycin antibiotics. This study therefore focuses on the microbiome associated with this single strain that has been in culture for over 70 years as a case study for further microbiome studies involving long-read sequencing. This proposed approach offers a more comprehensive, unbiased measure of bacterial diversity and metabolism in *A. carterae* cultures, both with and without antibiotic treatment.

*Amphidinium carterae* was chosen for this study firstly because of its cosmopolitan appearance in nature: it is one of the most common species found in sediments in multiple ecosystems [25,26,27]. *A. carterae* cultures are easy to maintain and can grow to relatively high densities compared to other dinoflagellate species [28,29]. This species is also often used as a model athecate photosynthetic peridinin-pigmented dinoflagellate due to a smaller genome compared to that of other free-living dinoflagellate species, and because it is a relatively early diverging toxic species [30].

In this study, we found populations of bacteria in an *A. carterae* culture that thrived in the presence of antibiotics, identified antimicrobial genes within these populations, and observed how the dinoflagellate culture responds when reverted back to antibiotic-free growth conditions.

## 2. Results

### 2.1. Bacterial Growth

**Figure 1:**
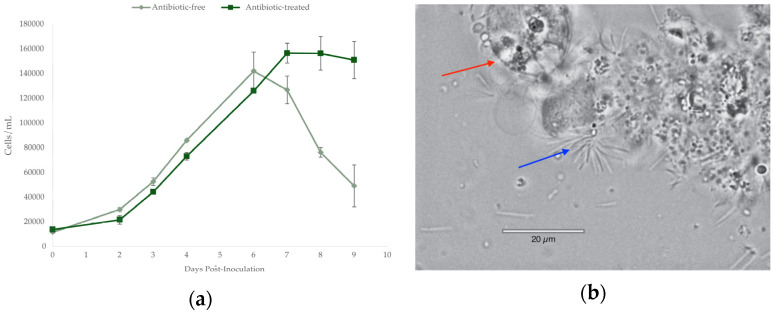
*Amphidinium carterae* growth with and without antibiotics. (**a**) Growth curves of *A. carterae* cultures; (**b**) image of lysed *A. carterae* (red arrow) taken at 100×, and the presence of rod-shaped bacteria (blue arrow).

The growth of *A. carterae* cultures grown both with and without antibiotics was found to be similar during both lag and log phases, with the daily maximum growth rate being about 63 ± 9% and 65 ± 9% for both treatments, respectively. Maximum cell densities were similar, with the antibiotic-free culture reaching 142,166 ± 15,382 cells/mL and the antibiotic-treated culture reaching 126,000 ± 1311 cells/mL. Growth differed between antibiotic and control treatments once the stationary phase began. In the antibiotic-treated culture, cell growth stagnated, and density remained stable, while antibiotic-free cultures showed significant decreases in cell number and signs of lysing and death under microscopy (Figure 1a). Cells within the antibiotic-free culture appeared to form aggregates, and dinoflagellate cells imaged at the beginning of the stationary phase in the antibiotic-free cultures displayed mass lysing and the appearance of rod-shaped bacteria overwhelming the culture (Figure 1b), alluding to algicidal properties. These bacteria were not observed by microscopy in the antibiotic-treated cultures.

### 2.2. Bacterial Identification

Metagenome assembly of *A. carterae* cultures identified 15 bacterial contigs based on 16S rDNA identity, ranging from 2.9 to 6.0 Mb (Table 1). All 15 were single contig genomes, and the *Flye* assembly reported 14 of the 15 being circular (all but *O. alexandrii*). The genome average depth of coverage ranged from 13 to 1612. Nearest neighbors of the 16S sequences found in these genomes were 98 to 100% identical to reference sequences in the Silva and GenBank databases (Supplemental Figure S1). The bacteria present included 10 Alphaproteobacteria, 1 Gammaproteobacteria, 3 Bacteroidia, and 1 Planctomycetota. Based on the very high (>98%) full-length 16S rDNA identity to previously described species, the names of these high-identity 16S rDNA matches are used as an operational taxonomy for the remainder of the text.

Within the 15 genomes, between 2890 and 5758 genes were predicted; of these, an average of 62 ± 13% had functional assignments according to BV-BRC, and of the hypothetical proteins, 74 ± 21% were longer than 100 amino acids (Supplemental Table S2). Genome quality was qualified as good for all of the genomes, with CheckM Completeness ranging from 77.5 to 100%, and the majority of the genomes showing above 90% completeness [31]. CheckM Contamination scores were below 5% for all the genomes as well.

**Table 1:** Bacterial genome characteristics.

NCBI Sample Title ^1^	Length	AT%
*Labrenzia* sp. strain ac12	6,053,707	40.9
*Hoeflea alexandrii* strain ac4	4,829,646	38.4
*Algihabitans albus* strain ac2	4,739,774	34.3
*Ahrensia marina* strain ac1	4,424,055	42.8
*Seohaeicola saemankumensis* strain ac14	4,275,009	36.6
*Roseovarius mucosus* strain ac13	3,969,289	38.9
*Rhodophyticola porphyridii* strain ac11	3,902,216	35.8
*Oceaniradius stylonematis* strain ac10	3,664,187	35.3
*Oceanicaulis alexandrii* strain ac9	2,992,841	37.7
*Nitratireductor* sp. strain ac15	2,917,504	39.8
*Marinobacter adhaerens* strain ac5	4,424,055	42.9
*Cyclobacterium xiamenense* strain ac3	5,806,256	51.6
*Marivirga tractuosa* strain ac6	4,787,102	65.3
*Muricauda* sp. strain ac8	4,366,883	58.0
Phycisphaeraceae SM1A02 strain ac7	3,415,114	34.9

^1^ 16S sequence identification found using both the SILVA ACT service and the NCBI BLASTn feature [32].

### 2.3. Bacterial Abundance

**Figure 2:**
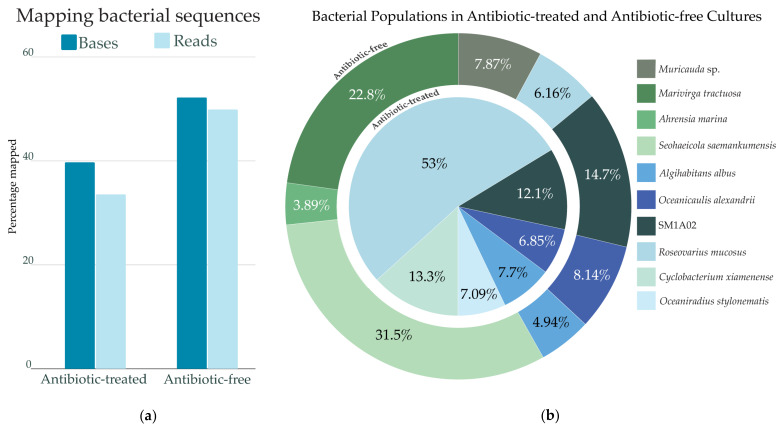
Bacterial populations with and without antibiotics. (**a**) Percent of reads and bases mapping back to bacterial genomes from both the antibiotic-free and antibiotic-treated cultures, aligned using minimap2. (**b**) Bacterial abundance based on read mapping in the antibiotic-free and antibiotic-treated cultures.

In the antibiotic-free cultures, 52.2% of the total bases and 49.9% of reads mapped to the 15 bacterial genomes, while in antibiotic-treated cultures, 39.8% of total bases and 33.5% of reads mapped to bacteria (Figure 2a). Ten of the 15 bacterial genomes identified accounted for 90% of the total bacterial reads mapped in both conditions (Figure 2b), and the remaining five taxa accounted for <10% of total bacterial reads.

All 15 bacteria were detectable at some level in all the batches of sequencing data, but the relative abundance differed substantially for seven bacterial genomes. Under antibiotic treatment, *R. mucosus* was the most abundant single bacterium (at 53%) and then decreased to 6.16% without antibiotics. Without antibiotics, *Muricauda* sp., *Marivirga tractuosa, Ahrensia marina,* and *Seohaeicola saemankumensis* rose to the top 90% of the bacterial reads. In the antibiotic-treated culture, *O. stylonematis* and *C. xiamenense* populations were among the top 90% of bacterial reads, but these bacteria were not abundant without the antibiotic treatment.

The antibiotic-free and antibiotic-treated cultures both included three taxa at roughly similar proportions: *SM1A02*, *O. alexandrii*, and *A. albus*. When cultures were grown without antibiotics, the abundance of *SM1A02* and *O. alexandrii* slightly increased by 2.6 and 1.3%, respectively, while that of *A. albus* slightly decreased by 2.8%. The remaining five bacterial genomes were present at low levels in both culture conditions.

### 2.4. Bacterial Metabolic Pathways

**Figure 3:**
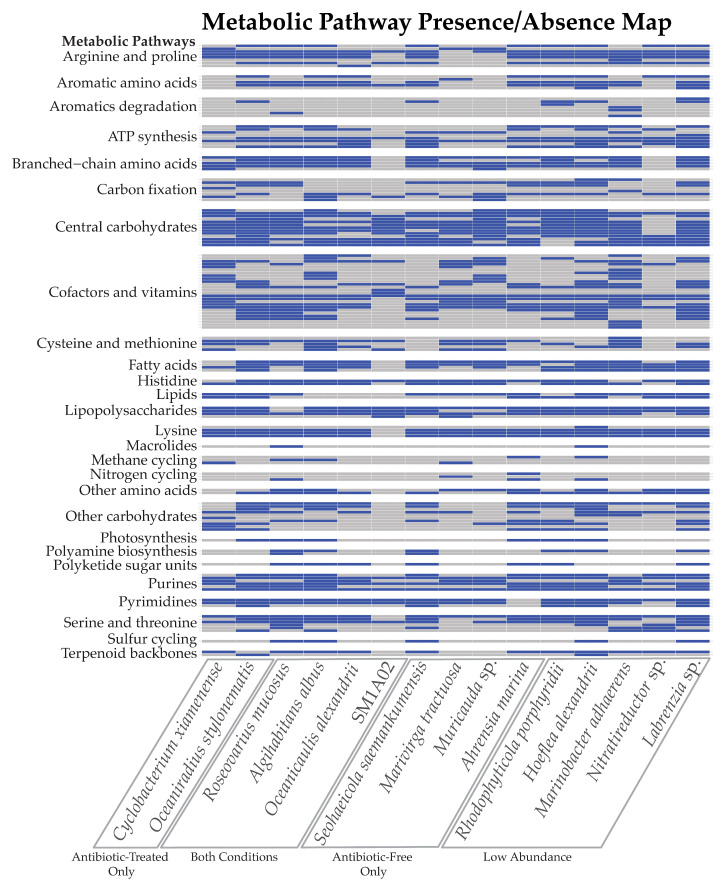
Functional traits identified via CARD based on the KEGG database [34]. Specific pathway names for each row are listed in Supplemental Table S3.

The metabolic abilities of the bacterial genomes were assessed using the KEGG database, showing a wide variety of potential pathways (Figure 3, Supplemental Table S3). Many of the bacterial genomes have potential pathways for the production of amino acids, such as arginine, proline, serine, and threonine. Basic metabolic pathways such as carbohydrate metabolism and ATP synthesis were identified. Some of the bacterial genomes, including *R. mucosus*, *R. porphyridii*, *H. alexandrii*, *O. stylonematis*, *A. marina*, and *A. albus*, were found to potentially contain photosynthesis machinery for photosystem II, as well as some carbon-fixation pathways. The *Labrenzia* sp. genome, although the largest bacterial genome assembled, was also in low abundance in the antibiotic-treated culture and contained a part of the reductive phosphate pentose cycle and the Calvin cycle, but not PSII.

Nitrate-reduction pathways were found in three of the bacterial genomes, and denitrification machinery was identified only in the *M. tractuosa* genome. The *Nitratireductor* sp., found in low abundance the antibiotic-treated culture, was negative for nitrate-reduction pathways despite the generic name, which aligns with prior research [33].

### 2.5. Secondary Metabolite Synthesis

**Figure 4:**
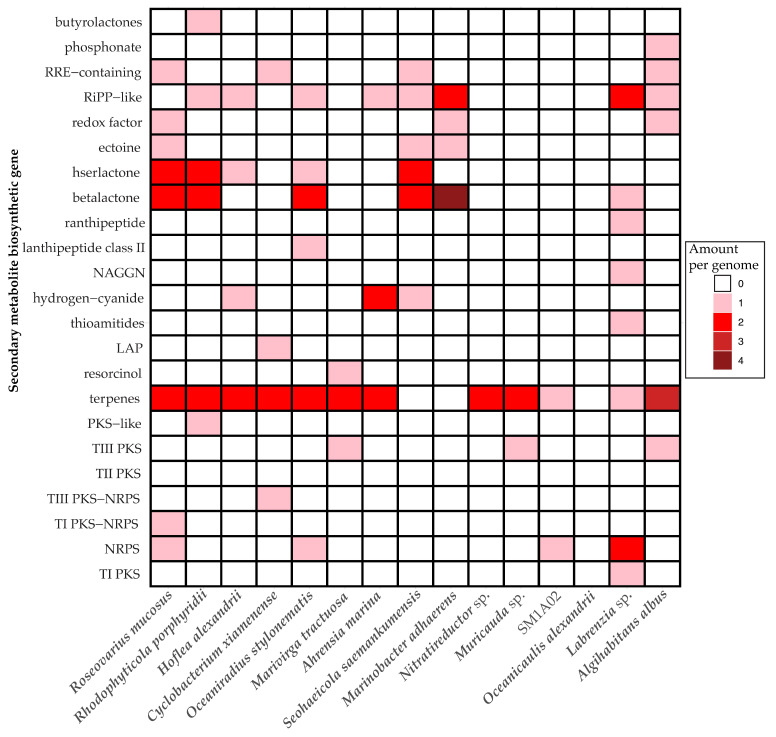
Secondary metabolites present in the bacterial genomes, identified by antiSMASH [35].

The assembled bacterial genomes were found to contain regions associated with the production of a variety of secondary metabolite compound classes, including building-block lactone structures such as butyrolactones, betalactones, and hserlactones (Figure 4). Terpenes and aromatic compound synthase genes were the major natural product occurring amongst the genomes. Some polyketide synthases (PKSs) and non-ribosomal peptide synthases (NRPSs) were identified, but none were predicted to participate in the biosynthesis of larger chemical structures according to antiSMASH.

### 2.6. Antibiotic Resistance

**Figure 5:**
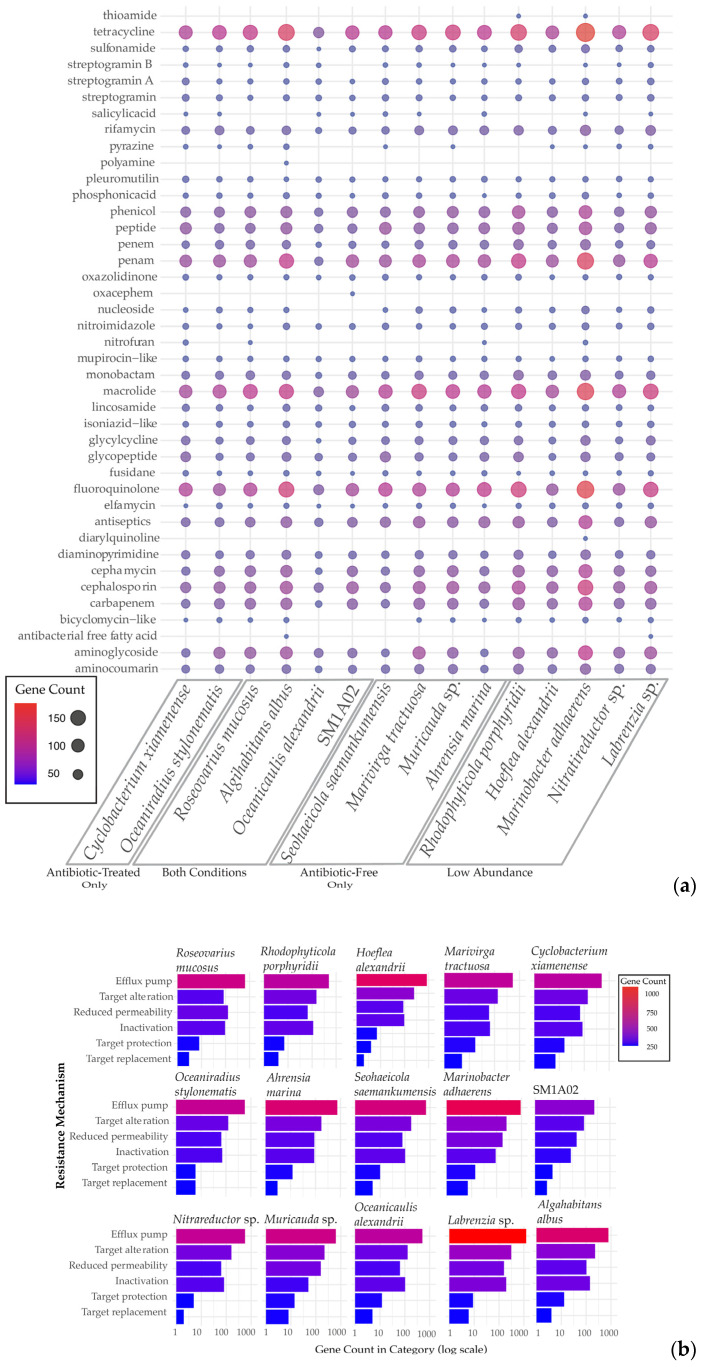
Antimicrobial resistance in assembled microbiome. (**a**) Antimicrobial resistance genes within each assembled bacterial genome (**b**) Specialty genes identified from the CARD database [36].

Generally, all of the bacterial genomes had antibiotic resistance genes in the CARD database search (Figure 5a). Interestingly, all of the genomes had a large number of resistance genes to tetracycline. The antibiotic resistance genes in the bacterial genomes found in the antibiotic-treated cultures did not appear to indicate a strong pattern of drug class resistance to the antibiotic cocktail used. Most of the antibiotic resistance genes were part of antibiotic efflux systems, especially in the assembled *Labrenzia* sp. genome (Figure 5b). This was generally followed by mechanisms including drug-target alterations, reduction of drug permeability, drug inactivation, drug-target protection, and drug-target replacement, as identified in the CARD database.

**Figure 6:**
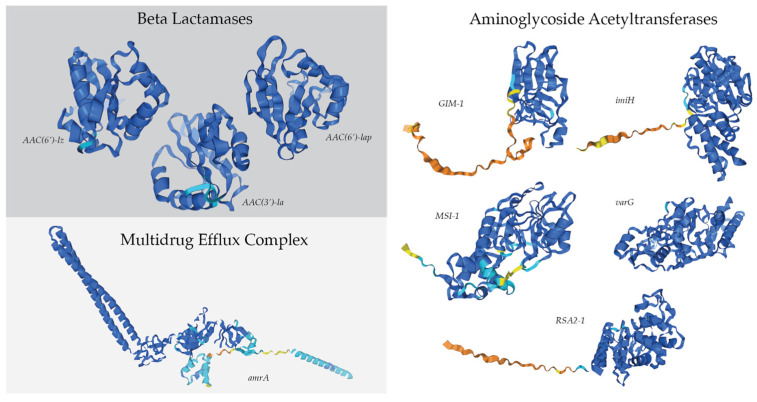
Antibiotic resistance genes’ protein structure predictions created by AlphaFold, unique to the assembled *Roseovarius mucosus* genome [37,38]. Confidence in structure ranged based on per-residue model confidence score (pLDDT) from dark blue (pLDDT > 90), light blue (90 > pLDDT > 70), yellow (70 > pLDDT > 50), to orange (pLDDT < 50).

The dominant bacterial genome in the antibiotic-treated culture, *R. mucosus*, contained 13 resistance genes that were either completely unique to the taxa or found only within bacterial genomes found in the antibiotic-treated culture (Supplemental Table S2). Of those 13, nine had the potential to confer resistance to carbenicillin, kanamycin A, and/or spectinomycin (Figure 6). Three of these genes encoded potential beta-lactamases, which inactivate beta-lactam antibiotics by hydrolyzing the peptide bond of the beta-lactam ring and may confer resistance to carbenicillin. One of the genes is similar to the efflux subunit of the AmrAB-OprM multidrug efflux complex, which impairs aminoglycoside accumulation. Lastly, five genes in the *R. mucosus* genome were identified as potential aminoglycoside acyltransferases, which may catalyze the AcCoA-dependent N-acetylation of amino groups on the aminoglycoside molecule (such as kanamycin A and spectinomycin).

## 3. Discussion

Among the 15 assembled genomes, every genome had a high (>95%) identity 16S rDNA match to an existing annotated sequence, suggesting that the culture did not contain any entirely novel bacteria. Many of the assembled genomes were identified as bacterial species that have previously been observed as co-existing with dinoflagellates. Interestingly, the assembled Planctomycetota bacteria were identified as *SM1A02*, an uncultured strain associated with many dinoflagellate cultures [39]. This species’ genome has previously been reconstructed using metagenomic assembly and binning. Research by Baker et al. found two of these *SM1A02* genomes to be 2.6 and 2.9 Mb, while we have assembled a 3.4 Mb genome here. Most recently, an assembly of *SM1A02* was produced from a *Karlodinium* culture, which agrees with our genome length of 3.4 Mb [40]. *SM1A02* is thought to likely be an anammox bacteria—efficient at nutrient removal, specifically through anaerobic ammonium oxidation [41]. This ability to oxidize ammonium to nitrogen gas may have an impact on the close association with dinoflagellate species.

*Roseovarius mucosus* was also identified, similar to a species found with the dinoflagellate *Alexandrium ostenfeldii*. In prior research, genes in *R. mucosus* were found that may play crucial roles in the interrelationship of the bacterium and dinoflagellate, such as genes for dimethyl sulfoniopropionate (DMSP) utilization. Research on the close interactions of DMSP-degrading *Roseobacter* species with DMSP-producing dinoflagellates is well-established [42]. Our metabolic results show that *R. mucosus* likely has pathways for thiosulfate oxidation, supporting these past findings [43]. The ability for thiosulfate oxidation may also have a connection to the common sulfation of toxic amphidinol products produced by *A. carterae* or more broadly for sulfur cycling within the cultures [44,45]. The pathway for assimilatory nitrate reduction found in the *R. mucosus* genome has been observed as a potential nitrogen source by some [46,47]. The pathway for polyamine biosynthesis for putrescine and spermidine has also been found in the *R. mucosus* genome, which may play an essential role in dinoflagellate growth [48,49,50]. The presence of PSII and other carbon-fixing pathways also aligns well with prior research of this species [51].

*Hoeflea alexandrii* and *Oceanicaulis alexandrii* were discovered with a dinoflagellate species of *Alexandrium* as well as bacterial species *Labrenzia alexandrii* and *Nitratireductor alexandrii*, which have high 16S identity to the *Labrenzia* and *Nitratireductor* species assembled here [52,53,54,55]. *Marinobacter adhaerens* has been found in close association with *Pyrodinium*, another toxin-producing dinoflagellate [56]. *Muricauda* species have been previously associated with *Amphidinium* as well [57]. A *Seohaeicola* species’ genome was recently assembled from a culture of *Karlodinium*, another toxin-producing dinoflagellate species [40]. Some species, such as *O. stylonematis*, *A. albus, C. xiamenense* and *R. porphyridii*, were discovered in association with other kinds of microalgae, such as diatoms and red algae [58,59,60,61,62].

Some species found only in the antibiotic-free cultures may be opportunistic due to the nutrient availability caused by dinoflagellate senescence. Both the assembled *R. porphyridii* genome and the *H. alexandrii* genome were found in very low abundances in the antibiotic-free culture. The *R. porphyridii* species is a genus of purple non-sulfur bacteria known to be halophilic and have the ability to perform photosynthesis [58]. Thiosulfate oxidation pathway genes were also found in the *H. alexandrii* genome.

The transition from antibiotic treatment to no untreated is unlikely to have resulted in the introduction of new bacteria to the culture, as all taxa were seen and could be at least partially assembled in sequencing of either antibiotic-treated or untreated cultures. Thus, the microbiome shift is more likely due to stronger growth of some species over others when antibiotics were present or absent rather than the recruitment of novel species during culture changes, which were performed under sterile conditions.

Previous studies have demonstrated that the use of the KAS-antibiotic treatment (with kanamycin A, ampicillin, and streptomycin) can be used to favor pigmented bacterial species [63]. Similar mechanisms may be why we saw such a shift towards bacterial populations with PSII and carbon fixation systems with the use of our antibiotic cocktail, from 12.5% to 50% of the highly abundant bacterial species having PSII pathways with the antibiotic-treatment. The *R. mucosus* identified in past dinoflagellate cultures, which dominated the antibiotic-treated microbiome in this experiment, has been shown to contain bacteriochlorophyll *a* [51]. There is also evidence of dinoflagellates protecting certain pigmented bacterial populations from antibiotics, as the pigmented bacteria may be protecting the microalgal cells from light stress via carotenoid production, which has previously been shown to be produced by multiple assembled bacterial species (Figure 4) [63]. In prior research regarding the coral symbiont dinoflagellate *Symbiodinium*, the bacterial microbiome was observed to support the dinoflagellate’s PSII yield and decrease the production or reactive oxygen species (ROSs) [64].

Antimicrobial resistance appears to be generally common amongst bacterial populations found in dinoflagellate cultures. In the case of the assembled genomes here, all of them had hundreds of potential antibiotic resistance genes that likely allowed their broad prevalence (Figure 5). The reasons for why some bacteria were found to survive better in antibiotic-treated or antibiotic-free conditions could be due to multiple causes. One may be that the minimum inhibitory concentration of the antibiotic used may differ from the actual concentration tested [65]. This may be due to the mechanism of resistance, or the antibiotic’s resistance to degradation (such as in the case of carbenicillin compared to ampicillin) [66]. The specific genes found within each of these resistance groups may have varying efficacy against the antibiotics as well. In the case of *R. mucosus*, which best-endured the antibiotic treatment used here, it may be that one of the nine antibiotic resistance genes had greater efficacy over one or more of the antibiotics used compared to the machinery found in the other genomes (Figure 6).

The extent to which the bacteria from the assembled genomes are mutual, commensal, or deleterious to the *A. carterae* population is still obscure. The fact that the dinoflagellate population significantly decreased and showed signs of mass lysis without antibiotics is circumstantial evidence that at least one abundant bacterial species in the antibiotic-free culture is likely the cause. Prior research on antibiotic effects on dinoflagellate growth has shown various results. In some cases, dinoflagellates appear to require their associated microbiomes to survive [11,67]. In the case of the antibiotic cocktail used here, the growth results align with previous observations of the antibiotic-treated *Amphidinium* cultures having a slightly extended growth phase and the ability to maintain higher densities of dinoflagellate cells [22]. Based on our microscopy analysis, we suspect the 10 µm length rod-shaped bacteria that began to accumulate around the start of mass cell lysis are likely harmful to the dinoflagellates and may possess some algicidal properties. We could deduce that the culprit may be *Marivirga tractuosa* or *Seohaeicola saemankumensis* due to the increased abundance found in the antibiotic-free culture (Figure 2b), as well as prior descriptions of this species as being rod-shaped. Cells of *M. tractuosa* can be between 10 and 50 µm long, while *S. saemankumensis* has been shown to be up to 5 µm in length [68,69,70]. The *M. tractuosa* genome lacks many main metabolic pathways, such as amino acid biosynthesis, suggesting that this species requires resources gained from the lysed dinoflagellate cells. The *M. tractuosa* genome has also been found to have complete denitrification pathways, which may contribute to a loss of bioavailable nitrogen in the culture [71]. The Coenzyme M pathway alludes to methanogenic abilities and production of methane, and potentially the use of dinoflagellate-released DMSP as a precursor [72,73]. Harmful algal blooms have been observed to precede methane increases in aquatic environments, which may be in part due to the shift in the microbial community [73].

Prior research has been contentious over the secondary metabolite synthesis potential of dinoflagellate microbiomes, and contrasting results have identified toxin production as a product either of the bacterial community or the dinoflagellate cells themselves [13,14,15,16,17,74,75,76,77]. The fifteen apparently complete genomes assembled from this culture likely represent the bulk of prokaryotic diversity due to the extent of our sequencing and the production of full, well-covered, complete genomes across a wide range of sequencing coverage and depth. Any missing diversity would likely be in very low abundance to evade detection and is not likely to be present at a level to contribute to toxin biosynthesis. Similarly, gene annotation provides a potentially complete picture of the culture metabolic potential. However, a large fraction of predicted genes was unannotated, likely due to imperfect prediction of protein coding genes as well as knowledge gaps of every possible bacterial pathway. Our genome analysis into secondary metabolite synthesis has shown no evidence of potential bacterial origin for a processive multidomain PKS gene responsible for the amphidinol toxins associated with our *A. carterae* culture. Since multidomain, processive PKS genes are very large open reading frames rich in easily defined conserved domains, these genes would be unlikely to have been missed in the genomes described here. Several multidomain PKS/NRPS genes derived from bacteria are present broadly across core dinoflagellate transcriptomes, which generally express a surfeit of domains associated with toxin production and lipid synthesis whether or not they are documented toxin-producing species [18]. More research could be done to see what effect metabolic pathways, such as thiosulfate oxidation, may have on toxin production. Although it seems most likely that *A. carterae* independently synthesizes amphidinols, the bacterial populations may contribute resources for the task, such as acetate [45,78,79]. Of the bacterial genomes assembled, only *M. adhaerens* was shown to have a complete pathway for a phosphate acetyltransferase−acetate kinase pathway, which produces acetate from acetyl-CoA (Figure 3). *Oceaniradius stylonematis, S. saemankumensis, R. porphyridii, H. alexandrii,* and the *Labrenzia* sp. all had complete phenylacetate degradation pathways to produce acetyl−CoA, which may serve as a precursor to acetate synthesis, and *O. stylonematis, S. saemankumensis, R. porphyridii, R. mucosus, A. albus, O. alexandrii, M. adhaerens,* and the *Labrenzia* sp. had complete pathways for leucine degradation to acetyl−CoA as well.

The microbiome of algal species has been shown to contribute necessary vitamins and products to dinoflagellate species, the most recognized being cyanocobalamin (B12) [80]. Based on our metabolic findings, the introduction of B vitamins into dinoflagellate growth media does not appear to be redundant with the biosynthetic abilities of the microbiome. The vitamins added to our ESAW media preparation include biotin (H), B12, and thiamine (B1) to increase growth rate and final yield [81]. Of the bacterial genomes assembled, only the *M. adhaerens* genome had a pathway identified to synthesize biotin, and this species was in very low abundance. The only highly abundant species with aerobic and anaerobic pathways for the synthesis of B12 were *R. mucosus* and *O. stylonematis*, which were significantly more abundant in the antibiotic-treated cultures, and their decline without antibiotics may have been a factor in the cell mortality of *Amphidinium* as the nutrients in the culture diminished over the log phase. Only *A. albus* within the highly abundant bacterial species had a pathway for vitamin B1 synthesis, and yet this was only through a salvage pathway that utilizes precursors or similar compounds in the surrounding media for B1 biosynthesis [82].

The Planctomycetota bacteria *SM1A02* is the only assembled genome in the *Amphidinium* culture to have full pathways for menaquinone (vitamin K2) biosynthesis. Although vitamin K1 is conventionally known as a redox cofactor in plants and green algae, vitamin K2 can also be a secondary electron acceptor of PSI in some algal and archaeal species [83]. Vitamin K2 can also shuttle electrons between different respiratory complexes in anaerobic respiration or aerobic respiration in a microaerophilic environment [84]. The effect of vitamin K2 bioavailability for dinoflagellate species remains to be seen.

## 4. Materials and Methods

### 4.1. Cell Culturing

*Amphidinium carterae* CCMP 1314 were grown in ESAW artificial marine media with a salinity of 20 ppt supplemented with f/2 nutrients without silicates at a starting concentration of 10 K cells mL^−1^ and allowed to acclimate for 14 days in a 14:10 h light–dark period in 100 μmol photons m^−2^ s^−1^ at 25 °C [81]. Cultures of *A. carterae* were provided by the National Center for Marine algae and Microbiota, initially isolated in 1954 from Nantucket Sound. The cultures have been continuously grown with an antibiotic cocktail of 100 μg/mL carbenicillin, 50 μg/mL kanamycin A, and 50 μg mL^−1^ spectinomycin (“antibiotic-treated”) for over a decade. For analysis, some cultures were weaned off growth in the presence of antibiotics over a month before subsequent DNA extraction (“antibiotic-free”). Cell counts were done using a Scepter 3.0 Cell Counter (Merck KGaA, Darmstadt, Germany) (Figure 1).

### 4.2. DNA Extraction and Long-Read Sequencing

For each dinoflagellate culture, the cells were spun down at 10,000× *g* for 10 min to form cell pellets. The cell pellets were then resuspended in 0.1 M EDTA and 0.5% SDS with 200 μg proteinase K and allowed to incubate for 10 min at 50 °C. Following this incubation, equal 16.6% volumes of 2% CTAB and 5 M NaCl were added, and once again the sample was allowed to incubate at 50 °C for 10 min. After this, the solution was mixed 1:1 with chloroform and allowed to sit at room temperature for 10 min. The sample was then spun for 10 min at 10,000× *g*, and the aqueous layer was transferred to a new tube and mixed with a binding buffer from the DNA Clean & Concentrator kit (Zymo Research, Irvine, CA, USA). Aliquots of 750 μL were spun through the columns, followed by washing and finally elution in 20–40 μL of water. Small fragments were filtered out using a PacBio SRE/SRE-XL kit (Pacific Biosciences, Menlo Park, CA, USA) according to manufacturer’s directions. A high-molecular-weight DNA library was then prepped using a Nanopore whole genome sequencing kit (SQK-LS114, Oxford Nanopore, Oxford, UK), followed by sequencing on a MinION, GridION, or PromethION device (Supplementary Table S1). Oxford Nanopore Technology with the “super” accuracy Dorado basecalling model was used due to its recent increase in basecalling accuracy of 99.5% [24].

### 4.3. Genome Assembly

Following sequencing, DNA data in pod5 files were basecalled by Oxford Nanopore’s Dorado basecaller (7.2.13) using the dna_r10.4.1_e8.2_400bps_sup@v4.3.0 model. Sequences from the antibiotic-free culture runs or antibiotic-treated runs were pooled and assembled using the Flye de novo assembler (2.9.1–b1780), filtering for sequences over 200 bases [85]. Bacterial contigs were identified using BLASTn for 16S sequences from NCBI’s 16S RefSeq or 16S microbial ribosomal databases. Bacterial matches from each assembly were identified, and duplicate matches between the antibiotic-free and antibiotic-treated runs were compared, and the most complete bacterial genomes were chosen for further analysis.

### 4.4. Phylogenetic Analysis

Once the bacterial genomes were identified, 16S sequences were extracted and identified using SILVA’s Alignment, Classification, and Tree (ACT) service according to the global SILVA alignment for rRNA genes, as well as through NCBI’s BLASTn feature [32]. A consensus of the two was chosen based on match percentage. A phylogenetic tree was constructed using RaxML including 10 nearest neighbors identified by SILVA ACT from the database (Supplemental Figure S1).

### 4.5. Read Abundance Mapping

To quantify the relative abundance of bacterial genomes within the antibiotic-free and antibiotic-treated cultures, reads from each condition were aligned to the single unified set of bacterial contigs using minimap2, using ordinary minimizers as seeds [86]. Alignment SAM files were converted to BAM using Samtools (version 1.16.1), then sorted, indexed, and finally coverage and stats files were created [87]. Genomes with greater than 90% coverage were considered present in the cultures, and abundance was calculated by the percent of reads mapping to that genome out of the total bacterial reads mapped in each condition.

### 4.6. Metabolic and Secondary Metabolites Analysis

Metabolic and biogeochemical functional trait profiles for each bacterial genome were assessed using METABOLIC-G using the KEGG database for pathway identification [34]. Genome sequences were also input into antiSMASH for secondary metabolite biosynthetic gene clusters search [35].

### 4.7. Antimicrobial Resistance Analysis

The Comprehensive Antibiotic Resistance Database (CARD) Resistance Gene Identifier (RGI version 6.0.3) software was used for the prediction of antibiotic resistance genes in the bacterial genomes [36]. Perfect, Strict, and Loose hits for genes were allowed, but partial genes were excluded. Protein predictions of antimicrobial resistance genes of interest were created using the AlphaFold Google DeepMind AI system [37,38]

## 5. Conclusions

Here we discussed the use of long-read sequencing to qualify the microbiome surrounding a dinoflagellate culture. With the extensive use of long-read sequencing, our results showed that we were successful in assembling full bacterial genomes that could be used to assess the functional qualities of the associated bacterial species. Although *A. carterae* may be grown without antibiotics, the viability of the culture and subsequent sequencing analyses may benefit from their use. The observed dying off of cells in the *A. carterae* cultures during the end of the log phase without antibiotics present may have been due to the proliferation of one of the more antibiotic-sensitive bacteria identified. We also found a decrease in the proportion of bacterial reads sequenced with the use of antibiotics. As novel sequencing technologies such as efficient long-read sequencing become more readily available, techniques to target sequences of interest will allow for more productive sequencing efforts. The data suggest that both comprehensive and quantitative microbial genome sequencing can be accomplished from this culture, which could in the future be expanded to work with in situ sequencing of dinoflagellate blooms.

## Data Availability

All of the raw sequence and assembled data have been deposited under the NCBI project number SUB14439053 and SRA numbers SRR28996185, SRR28996184, SRR28996183, SRR28996182. GenBank Accession numbers for the bacterial genomes are CP159469 through CP159483 (Table S3).

## Supplementary Materials

The following supporting information can be downloaded at: https://www.mdpi.com/article/10.3390/md22080342/s1, Figure S1: 16S Phylogenetic Tree; Table S1: Sequencing Runs; Table S2: Basic Stats on Bacterial Genomes; Table S3: METABOLIC Pathway Identifiers; Table S4: *Roseovarius* Unique AMR Genes.
