# Supplementary material for: Long-Read Sequencing Unlocks New Insights into the Amphidinium carterae Microbiome
Source: Mar Drugs. 2024 Jul 27;22(8):342. doi: 10.3390/md22080342 (PMC11355691; doi:10.3390/md22080342)
Supplement: Supplementary file 1 [file marinedrugs-22-00342-s001.zip › Figure S1.pdf]

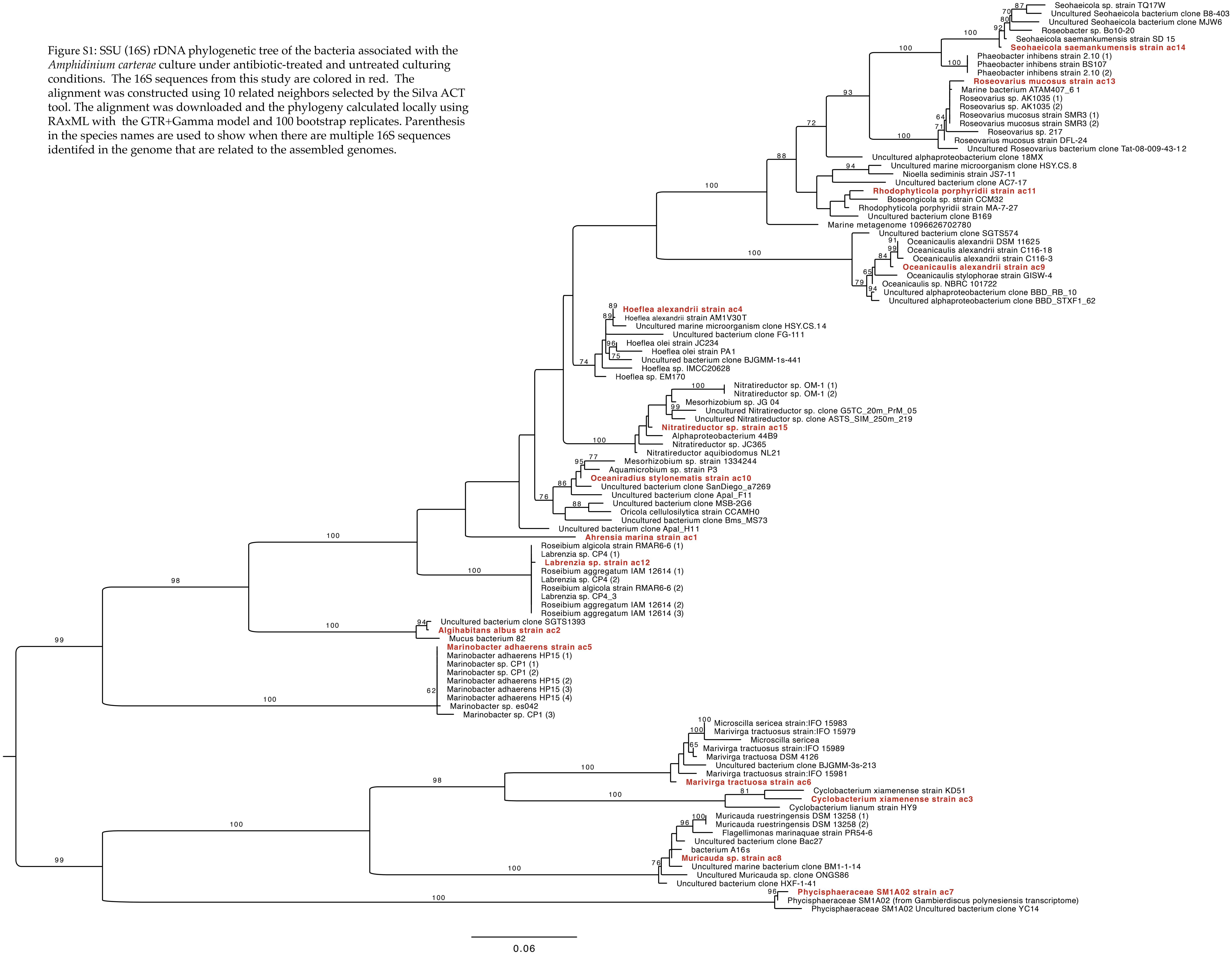

Figure S1: SSU (16S) rDNA phylogenetic tree of the bacteria associated with the *Amphidinium carterae* culture under antibiotic-treated and untreated culturing conditions. The 16S sequences from this study are colored in red. The alignment was constructed using 10 related neighbors selected by the Silva ACT tool. The alignment was downloaded and the phylogeny calculated locally using RAxML with the GTR+Gamma model and 100 bootstrap replicates. Parenthesis in the species names are used to show when there are multiple 16S sequences identifed in the genome that are related to the assembled genomes.
